# Evaluation of a text message + pedometer intervention to increase steps after emergency department discharge: a pilot study

**DOI:** 10.1007/s40520-025-03030-7

**Published:** 2025-04-21

**Authors:** Brian Suffoletto, Waverly Mayer, Caitlin Toth, Nick Ashenburg, Michelle Lin, Michael Losak, David Kim

**Affiliations:** Department of Emergency Medicine, Stanford University, Stanford, USA

**Keywords:** Older adults, Fall prevention, Emergency department, Physical activity, Text message, Pedometer, Mobile health, Behaivor change, Step count

## Abstract

**Background:**

Older adults face increased risk of functional impairments after Emergency Department (ED) discharge, yet no evidence-based interventions exist for this population.

**Objective:**

To evaluate the feasibility, safety, and effectiveness of Safe Steps, a text message + pedometer intervention designed to motivate individuals to meet step count goals.

**Methods:**

We recruited ED patients aged ≥ 60 with low physical activity. Participants received a pedometer in the ED, daily text messages to report steps, and weekly prompts to set step goals over 4 weeks. We assessed step count reporting rates, falls, and change in steps over time.

**Results:**

Among analyzed participants (*n* = 40), daily step reporting was high (95% of weeks with 2 + readings). No participants had falls due to the intervention. Step count increased by a mean of 359 steps per week (95% confidence interval 182–536).

**Conclusion:**

Safe Steps appears feasible, safe, and may be effective for promoting activity after ED discharge.

**Supplementary Information:**

The online version contains supplementary material available at 10.1007/s40520-025-03030-7.

## Background

Older adults account for over 20 million annual Emergency Department (ED) visits [1], with nearly 25% experiencing functional decline within 30 days post-discharge [2]. Functional decline is often linked to physical inactivity, which modest increases in daily walking can mitigate [3]. Despite expert panels recommending physical activity interventions after hospital discharge [4], participation in group-based programs are notoriously low [5]. While wearable activity trackers paired with digital behavioral interventions delivered through ubiquitous technology like text messaging [6] could overcome participation barriers and have been shown to increase step counts among older adults [7], to our knowledge none have been tested in the post-acute care period. This study evaluates Safe Steps, a text + pedometer intervention tailored to older adults discharged from the ED. Using behavior change techniques such as self-monitoring and goal setting, the intervention aims to increase motivation for achieving daily step count goals. The primary objectives were to assess the intervention’s feasibility and safety, with a secondary focus on preliminary effectiveness. We hypothesized that Safe Steps would produce increases in mean step count over time.

## Methods

This single-center, single-arm pilot study was approved by the institutional review board. A sample size of 40 participants was chosen based on recommendations for pilot studies when the standardized effect size is small (0.2) to medium (0.5) and to assess feasibility metrics [8]. Eligible participants were ED patients aged ≥ 60 years reporting low physical activity (< 7,500 steps/day or < 75 min/day walking) who could walk unaided and consent to participate. Exclusion criteria included living in a nursing home, limited English proficiency, or inability to provide informed consent.

### Safe steps intervention

Safe Steps employs behavior change strategies, including self-monitoring and goal setting, to encourage older adults to increase daily step counts while maintaining autonomy. Participants received daily text prompts to report steps and tailored feedback based on step count thresholds, along with weekly goal-setting prompts, which were later modified to enhance participant autonomy and adherence. A description of the development, content, flow diagrams and sample messages are included in **Supplemental materials**.

### Study procedures

Research associates (RAs) approached medically stable ED patients after assessing eligibility with their ED physician. Once eligibility criteria were confirmed and the patient demonstrated that they could walk safely and informed consent obtained, the RA provided participants with a 3DFitBud pedometer (3DActive.com) in the ED and basic instructions on use. This device was selected for its accuracy [9] and large digital display suitable for older adults with visual impairments. At baseline, the RA collected information on demographics, ED chief complaint, prior falls, mobility, activity history, and barriers to routine physical activity to assess as potential covariates in engagement and intervention response. Full description of baseline assessments are included in **Supplemental materials**. At 30 days post-enrollment, the RA assessed falls during the study period—focusing on whether participants perceived the fall was related to the Safe Steps intervention. Safety was assessed by asking if participants ever felt unsafe increasing their step count (with response options of yes or no), with a prompt for description if they answered yes. Participants rated the intervention’s motivational impact on a scale from 0 (completely unmotivating) to 10 (highly motivating).

### Data analyses

Feasibility was assessed by analyzing the percentage of weeks with at least two days of daily step count reports and weekly goal-setting rates. Safety was assessed by analyzing falls attributable to increasing step count and perceived safety when walking. Effectiveness was assessed by analyzing changes in weekly step counts using a mixed-effects model. Putative mechanism was explored by analyzing perceived effect of intervention on motivation. A full description is included in **Supplemental materials**.

## Results

### Study enrollment and retention

Between January and May 2024, we screened 170 participants from an ongoing observational fall study. Of these, 61 (35.9%) met our low physical activity criteria, and 43 (71.6%) enrolled in Safe Steps. Three participants (7.0%) withdrew due to pedometer loss (*n* = 2) or too busy (*n* = 1) resulting in 40 retained participants who were included in analyses. We achieved high completion rates for follow-up assessments: 95.0% (*n* = 38) for fall assessment and 92.5% (*n* = 37) for effectiveness and safety measures.

### Participant characteristics

The study cohort (mean age 70 years) was predominantly White (75%) with balanced gender distribution (50% female). While only 15% reported falls in the previous three months, nearly half reported near-falls, unsteady gait, and positional dizziness. Most participants (75%) reported walking less than one hour daily. Few participants cited barriers to physical activity, with health concerns (17.5%) and injury fears (10%) being most frequent. See Table 1 in **Supplemental materials** for participant characteristics.

### Feasibility

Out of 160 weeks of data (40 participants x 4 weeks), 100 weeks (62.5%) had no missing days. 152/160 weeks (95%) had the minimum two days step count reports. No increases in missingness occurred over the 4 weeks. Daily step count reporting adherence for each week is shown in Fig. 1 in **Supplemental materials**. Missing data stemmed from unworn pedometers (57.5%) or unreported evening steps (42.5%). Weekly goal-setting, which was prompted at the end of week one through three, improved from 37.5% in week one to 52.5% in week three, with 37.5% of participants completing at least two weekly step goals.

### Safety

Three participants (7.5%) reported falls during follow-up, with two sustaining minor injuries. No falls were attributed to increased walking activity, and fall risk showed no association with step counts or activity progression. Two participants reported safety concerns: one due to calf strain and another from balance issues.

### Effectiveness

**Fig. 1:**
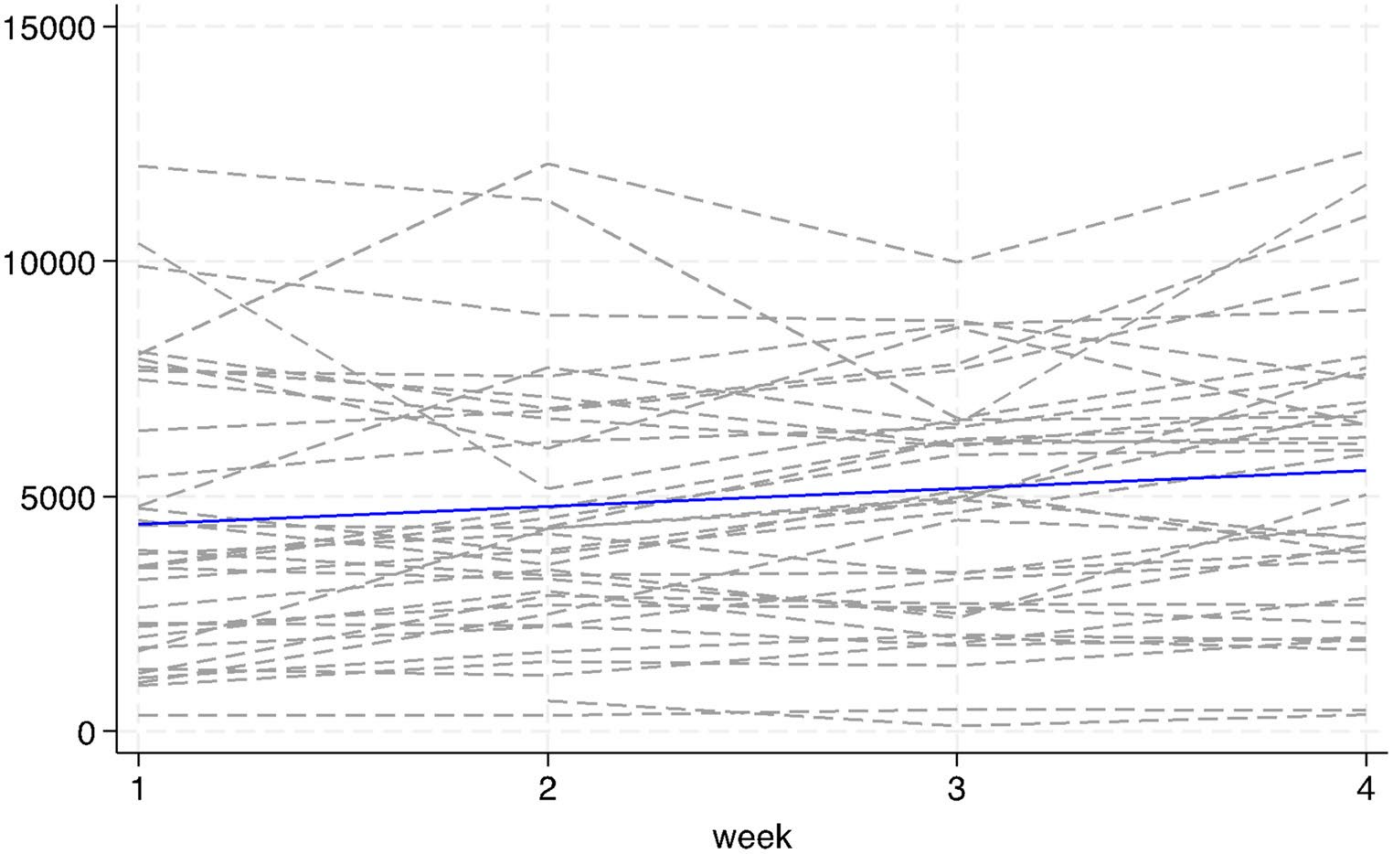
Step count trajectories. Dotted black lines represent individual weekly means for each participant. Solid blue line represents the fitted regression slope from the mixed effects regression

As shown in Fig. 1, the mean step count in the first week was 3994 steps/day and increased by 359 steps per week (95% CI: 182 to 536, *p* < 0.001). There was significant variability in baseline step counts across participants. Separately, at follow-up, participants (*n* = 37) rated the intervention highly motivating (mean = 7.4/10; Standard Deviation (SD) = 2.5).

## Discussion

This study demonstrates that a text-message + pedometer intervention is feasible, safe, and may be effective for increasing physical activity among older adults after ED discharge. Building on prior research showing that digital health interventions [10] and wearable activity monitors [7] can be used to promote physical activity among older adults, our findings suggest that a simple, remote intervention leveraging low-cost technology may provide an accessible way to support mobility during the post-acute care period, a high-risk time for functional decline.

Several key insights emerged from this pilot study. First, adherence to step reporting was high with over 95% of participants providing at least two step count values per week. Missing data due equally due to forgetting to wear the pedometer and failing to respond to end-of-day text prompts, suggesting that daily text messaging and pedometer step tracking are feasible and well-suited to older adults’ capabilities. Second, while weekly step goal prompts were met with moderate engagement, further design refinements may be needed to enhance participation with goal setting, even with our revised approach emphasizing autonomy.

Encouraging physical activity in older adults, particularly those at risk of falls, presents a unique challenge—balancing the well-documented benefits of movement with concerns about injury. *Safe Steps* was well-tolerated, and no falls were attributed to increased activity. However, two participants reported safety concerns, highlighting the importance of individualized pacing and self-monitoring in behavior change interventions. Future studies should explore personalized step goals and fall prevention strategies while maintaining the autonomy and accessibility that make remote interventions appealing.

*Safe Steps* produced a modest but statistically significant increase of approximately 360 steps per week. While even small increases in daily activity have been linked to health benefits in older adults [11, 12], the clinical significance of this step count increase remains uncertain. Additionally, participants found the *Safe Steps* intervention highly motivating, aligning with our hypothesized mechanism of addressing motivational barriers to physical activity. The incorporation of behavioral strategies to influence other barriers of physical activity (e.g. fear of falling) could amplify effects. Expanding the intervention to target other barriers to mobility, such as fear of falling, may enhance its impact.

Despite promising findings, this study has limitations. The single-center design and convenience sampling may limit generalizability, and inclusion criteria requiring smartphone ownership may have introduced selection bias. Additionally, we did not assess cognitive impairment, which could have influenced intervention engagement. Self-reported data and 8 PM step reporting prompts may have led to underestimation of true step counts. Quantitative measures may not have captured additional important barriers and enablers to the intervention. Finally, the short follow-up period precluded assessment of long-term behavior change.

Future research should purse testing scalable interventions like *Safe Steps* against a suitable comparator, assess multiple health and quality of life outcomes, and determine the longer-term impact of *Safe Steps* on older adults after ED discharge. Given the accessibility of text messaging and pedometers, this approach has strong potential to bridge the gap in post-acute mobility interventions for older adults.

## Electronic supplementary material

Below is the link to the electronic supplementary material.

Supplementary Material 1

## Data Availability

De-identified participant data that underlie the results reported in this article will be made available to researchers who provide a methodologically sound proposal. Data will be available beginning 3 months and ending 36 months following article publication. Proposals should be directed to suffbp@stanford.edu; to gain access, data requestors will need to sign a data access agreement.
