# Supplementary material for: Evaluation of a text message + pedometer intervention to increase steps after emergency department discharge: a pilot study
Source: Aging Clin Exp Res. 2025 Apr 21;37(1):131. doi: 10.1007/s40520-025-03030-7 (PMC12011956; doi:10.1007/s40520-025-03030-7)
Supplement: Supplementary file 1 — Supplementary Material 1 [file 40520_2025_3030_MOESM1_ESM.docx]

**Supplemental Material**

1. **Development of the Safe Steps Intervention**

The development of the *Safe Steps* text message intervention was informed by existing literature on barriers to physical activity among older adults^1^, which highlight concerns about health and fitness and lack of motivation as key drivers. Contextual barriers specific to the post-acute care discharge period, such as feeling unwell or managing acute conditions, further hinder engagement in regular walking. To address these challenges, *Safe Steps* incorporated evidence-based behavioral techniques—particularly self-monitoring and goal setting—both of which have been shown to effectively increase physical activity in older adults^2^.

Our team leveraged over 20 years of experience designing effective SMS-based interventions across a range of health behaviors, including hypertension management^3^ and medication adherence^4^, to ensure the intervention was both engaging and feasible for older adults. Initial intervention concepts were refined through discussions with a geriatric patient advocate and emergency medicine (EM) stakeholders, ensuring that messages were appropriate, motivating, and considerate of the unique needs of older adults recovering from an ED visit. We then collaborated with Stanford Research IT to integrate the intervention into REDCap using Twilio’s API, enabling automated, interactive text messaging. Finally, we fine-tuned the message scheduling protocol to optimize engagement, balancing the frequency and timing of step prompts and goal-setting reminders to support sustained participation.

1. **Description of Safe Steps**

Safe Steps uses behavior change strategies (i.e. self-monitoring and goal setting) previously shown to effectively enhance physical activity^5,6^ and provides tailored suggestions to guide individuals toward increasing their daily steps while maintaining autonomy and choice^7,8^.

Daily end-of-day messages focused on raising awareness of step counts and encouraging daily physical activity. Daily feedback was tailored based on evidence-based step count thresholds for older adults^9^. Daily end-of-day text prompts at 8 PM asked participants to report pedometer-measured steps, followed by tailored feedback for three threshold levels (>5,000; 2,000–5,000; <2,000 steps) based on evidence-based step count thresholds for older adults^9^.

During weeks 1-3, participants who provided four or more days of step data received a weekly summary report and goal-setting prompt at 2 PM each Sunday, as prior research has established that a minimum of four days provides reliable estimates of weekly physical activity patterns in older adults^10^. After enrolling 20 participants, we modified the goal-setting protocol due to low adherence to the preset weekly targets (only 45% of participants setting goals aligned with recommendations). The original protocol suggested specific step count increases (e.g., "Consider setting a goal of X steps above your average"). The revised protocol emphasized participant choice (e.g., "Based on your average of X steps, what goal would you like to set for next week?"). This modification aligned with self-determination theory principles supporting autonomy in behavior change^8^.

1. **Baseline assessment descriptions**

Fall history questions were taken from the CDC STEADI tool^11^. Mobility was assessed using an EQ-5D^12^ item, where participants rated difficulty with walking on a 4-point scale. We used items from the Community Healthy Activities Model Program for Seniors (CHAMPS) Physical Activity Questionnaire for Older Adults^13^ to asses typical walking time per day and other strenuous activity. Barriers to routine physical activity were measured by agreement with seven potential reasons not to exercise^14^ with responses on a 5-point scale collapsed into a dichotomous variable of agreement if participant agreed somewhat or agreed completely with a statement.

1. **Statistical Analyses**

Feasibility of daily step reporting was assessed by calculating the proportion of days participants provided at least two step count readings per week, based on prior research indicating this threshold provides valid estimates^15^. Missing step count data were classified as either (1) unworn pedometers (recorded as 0 steps) or (2) non-response to text messages. Feasibility of weekly step goal-setting prompts was evaluated through (1) goal submission rates and (2) the step count goals participants set. Safety outcomes included fall incidence, participant attribution of falls to increased activity, and self-reported safety concerns. Effectiveness was assessed by examining changes in step count over the intervention period. Weekly step counts were averaged for each participant when at least two readings were available; otherwise, the week was coded as missing. A linear mixed-effects model was used to evaluate step count changes over time, with week as the primary predictor and random intercepts to account for individual baseline variability. The model was estimated using Restricted Maximum Likelihood (REML), a standard approach for variance estimation in mixed models. To explore potential mechanisms, we analyzed participants’ perceived effect of the intervention on motivation. All analyses were conducted using Stata 14.0 (StataCorp, College Station, TX).

1. **Message Flow of Safe Steps**

1. **Safe Steps Messages Libraries**

Low [0< daily step count <= 2000]

- Small steps every day can lead to big improvements in health. Proud of you for starting this journey.
- Did you know that walking daily can lower the risk of heart diseases and improve mental well-being? Your health is important!
- We noticed you're starting on your step journey. Remember, it's not about the count but the consistency. Keep going!
- Every step you take is a step towards better health. You're doing great, and we're here cheering you on.
- We know that pain can make it difficult to be as active as you'd like. Studies show that being active can reduce pain.
- Staying inactive can also affect your mental well-being, increasing risks of depression and anxiety. Walking daily can boost your mood!
- Walking doesn't have to be a chore. Listen to your favorite music or an audiobook. It can make the experience enjoyable and motivating.
- Challenge yourself, but always listen to your body. You're doing great!
- Consistency is key. Even if it's a few more steps each day, that progress adds up. You've got this!
- We all have our pace, and that's okay. Celebrate every step you take. You're doing wonderfully!
- Every step counts, literally! Even if 2,000 steps sound daunting, remember it's just a series of small walks. You can do this!
- Turn chores into steps! Need to vacuum or sweep? It’s a great way to add some steps and keep your home clean.
- If evenings are better for you, how about a gentle walk to wind down your day? Listen to your body and move when it feels right.
- Experiencing discomfort or pain while walking is valid. Start slow and consider short, gentle strolls. ***Please consult a doctor if the pain continues***. Your well-being is paramount.
- Practice balance exercises. They'll improve stability and help you walk confidently.
- If walking causes discomfort, try wearing comfortable shoes or consider a walking aid. It's important to be safe and comfortable.
- Setting a gentle reminder or scheduling a walk at the same time daily can help make it a routine. Remember, even a short stroll around the house counts.
- Notice when your body wants a movement break. Stand up, wiggle your toes, stretch those legs, and discover what's around you. A few minutes of movement can make a big difference.
- Pairing up with a walking buddy can be a fun way to remember to take your walks. It's a great way to socialize and stay active together.
- Consider joining a balance or gentle exercise class. It can help improve stability and reduce the fear of falling. Your safety is essential.
- TV time can be active time! Walk in place or do simple exercises during shows.
- Walking is also a social activity. Invite a friend or join a local walking group. It's a wonderful way to connect with others while prioritizing your health.
- It's natural to be concerned about falls. Using non-slip shoes and walking in well-lit areas can boost your confidence. Remember, it's okay to use a walking aid for extra support.
- Your dedication is admirable, and we're cheering you on. Try to achieve more than 2000 steps per day. We believe in you.
- Your intention sets the course. Create goals that motivate you and let your determination propel you forward. We're here to support you.
- Each milestone crossed is a reminder that success is built one step at a time. Celebrate your progress and keep moving forward!
- Remember, it's not about the distance but the dedication. Keep taking those steps, no matter how small.
- Having a step goal should feel motivating. Begin with a number that feels comfortable for you, and remember, it's okay to adjust as you go along.

Moderate [2000< daily step count <= 5000]:

- Fantastic job today! Your steps today are a testament to your dedication. Keep up the great work!
- Well done! Every step you took today contributes to your overall health and well-being. We're proud of your efforts.
- You're making steady progress with your steps! Remember, consistency is key. Keep it up!
- You did wonderfully today! Those steps are not just numbers; they're steps towards better health.
- Congratulations on your steps today! It's clear that you're putting in the effort, and it's paying off.
- You're on a fantastic voyage to better health. Keep exploring with every step!
- Look at you go! Today's step count is a great achievement. Celebrate these moments of progress.
- Every day is a new opportunity to work on your fitness. You got this!
- Your step count today is an inspiration. You're proving that every step counts. Keep moving forward!
- You're doing amazing things for your health with every step. Today's count is proof of your resolve. Way to go!
- Your steps today are a great balance of effort and care. You're doing a remarkable job in maintaining this momentum. Cheers to you!
- Incredible work getting some walking in today! Keep those steps coming!
- You're making impressive strides on your health journey!
- You're proving that progress comes with persistence. Nice work!
- Celebrate your daily wins! Today was a day of good movement, keep it going tomorrow.
- You're walking your way to a healthier you, keep it up!
- Nice work! Consistency can be your strength – keep stepping towards a better future.
- You're turning small steps into significant achievements. The results are impressive
- Every step you take is a victory on your journey to better health.
- Your step count is the measure of your determination. Celebrate the small wins on your path to greatness!
- Your steps are the building blocks of a stronger you. Keep constructing!
- Each and every step is progress on your movement journey. Keep it up!
- Your steps are a reflection of your commitment to improving your health. Don't lose your forward momentum.
- With every step, you're writing your own success story. Keep walking and celebrating your journey!
- Impressive progress on your steps – keep striding to success!
- Nice work today! Raise the bar and let your step count soar.
- Boost your energy, mood, and health – one step at a time.
- Step up, step out, and celebrate the journey of a thousand steps. Each one counts toward your well-being!

High [daily step count > 5000]

- Outstanding effort! Your step count today is truly commendable. Here's to many more active days like this one.
- Good job walking. Keep it up!
- You are on the move today! Keep the momentum going tomorrow.
- What a milestone! Your high step count today is a testament to your perseverance and drive. Celebrate this achievement.
- These steps are essential to your health and wellness. Good job!
- Nice! Great step count!
- You've outdone yourself today! It's so inspiring to see your commitment in action. Bravo!
- Your walking is amazing – continue stepping towards your goals!
- You're on fire! Today's step count is a brilliant reflection of your dedication to staying active. We're incredibly proud of you.
- Hats off to you! Recording such a high step total is no small feat. Your hard work and determination are paying off.
- Your efforts today are nothing short of amazing!
- Keep reaching for the stars with your steps. We're cheering you on every step of the way.
- Your commitment to staying active is truly motivating. Continue the exemplary work and watch your progress unfold.
- Fantastic job on achieving this high step count today. Your dedication to health and wellness is truly inspiring.
- Impressive work on those steps! Maintain that momentum!
- Your strides today have been outstanding! This high step count is evidence of your commitment and effort. Carry on with the excellent work!
- Keep walking, keep achieving – you're doing great!
- ​You are movin’ and groovin’! Nice work!
- Continue the journey! Walking may be simple, but it's a powerful way to improve your well-being
- Today's step count looks great. Way to go!
- Kudos on your walking! Stay the course and remember to celebrate your fitness wins.
- You're crushing it on daily walking. Strut those steps!
- Consistency rocks, and so do you! Let those steps grooving!
- Impressive step count! Strut your stuff and maintain an active lifestyle.
- You've built a great habit of staying active. Push forward, and you'll observe more positive changes in your energy, fitness, and overall well-being.
- You did great today! Keep on stepping to even greater heights!
- Your determination and efforts have paid off. Beautiful job!
- You did it! Celebrate this success and continue working toward your next milestone!

1. **Table 1: Participant Characteristics**

|  | **Enrolled (n=43)** | | **Analyzed (n=40)** | |
| --- | --- | --- | --- | --- |
| **Variable** | **Mean/N** | ***SD/%*** | **Mean/N** | ***SD/%*** |
| ***Demographics*** |  |  |  |  |
| **Age** | 70 | *6.1* | 70 | *6.3* |
| **Sex** |  |  |  |  |
| Female | 21 | *49%* | 20 | *50%* |
| Male | 22 | *51%* | 20 | *50%* |
| **Race** |  |  |  |  |
| Caucasian/white | 33 | *77%* | 30 | *75%* |
| Black | 4 | *9%* | 4 | *10%* |
| Hawaiian/Pacific Islander | 2 | *5%* | 2 | *5%* |
| Asian | 2 | *5%* | 2 | *5%* |
| Multiracial | 2 | *5%* | 2 | *5%* |
| **Ethnicity** |  |  |  |  |
| Hispanic/Latino | 1 | *2%* | 1 | *2.50%* |
| **Marital status** |  |  |  |  |
| Single | 7 | *16%* | 6 | *15%* |
| Married | 26 | *61%* | 25 | *62.50%* |
| Separated/ Divorced | 4 | *9%* | 4 | *10%* |
| Widowed | 6 | *14%* | 5 | *12.50%* |
| **Living situation** |  |  |  |  |
| Self | 8 | *19%* | 7 | *17.50%* |
| Others | 35 | *81%* | 33 | *82.50%* |
| ***Chief complaint category*** |  |  |  |  |
| Abdominal/Digestive | 5 | 11.60% | 4 | *10%* |
| Chest pain | 7 | 16.30% | 7 | *17.50%* |
| Shortness of breath | 4 | 9.30% | 4 | *10%* |
| Pain | 4 | 9.30% | 4 | *10%* |
| Dizziness/Weakness | 8 | 18.60% | 7 | *17.50%* |
| Vision issue | 1 | 2.30% | 1 | *2.50%* |
| Leg swelling | 2 | 4.70% | 2 | *5%* |
| Fever | 1 | 2.30% | 1 | *2.50%* |
| Other | 11 | 25.60% | 10 | *25%* |
| ***Fall History*** |  |  |  |  |
| Any fall in the past 3 months | 6 | *14%* | 6 | *15%* |
| Any near-fall past 3 months | 20 | *46%* | 20 | *50%* |
| Feels unsteady when walking | 19 | *44%* | 17 | *42.50%* |
| Feels dizzy when standing (at least sometimes) | 21 | *49%* | 21 | *52.50%* |
| **Typical walking per day** |  |  |  |  |
| <30 minutes | 15 | *36%* | 15 | *37.50%* |
| 30 min to 1 hour | 16 | *38%* | 15 | *37.50%* |
| 1 to 2 hours | 8 | *19%* | 7 | *17.50%* |
| >2 hours | 3 | *7%* | 3 | *7.50%* |
| **Typical time with other strenuous PA** |  |  |  |  |
| None | 14 | *33%* | 12 | *30%* |
| <30 minutes | 12 | *29%* | 12 | *30%* |
| 30 min to 1 hour | 8 | *19%* | 8 | *20%* |
| 1 to 2 hours | 5 | *12%* | 5 | *12.50%* |
| >2 hours | 3 | *7%* | 3 | *7.50%* |
| **Barriers to routine physical activity** |  |  |  |  |
| No time | 3 | *7%* | 3 | *7.50%* |
| Afraid I will get hurt | 4 | *9%* | 4 | *10%* |
| No activity partner | 3 | *7%* | 3 | *7.50%* |
| Health reasons | 7 | *16%* | 7 | *17.50%* |
| No appropriate exercise programs | 2 | *5%* | 1 | *2.50%* |
| Lacks transportation | 0 | *0%* | 0 | *0%* |
| Not interested | 2 | *5%* | 2 | *5%* |

**Table 1 Legend:** Other chief complaints included: Animal Bite, Ankle Swelling, Bicycle Crash, Diverticulitis, Hypertension; Medication Refill, Rash, Visual Disturbance. Fall history measures were taken from the CDC STEDI checklist. Physical activity measures were taken from the CHAMPS Physical Activity Questionnaire for Older Adults.

1. **Figure 1: Completion of Daily Step Count Reports by Week**

**Supplement Bibliography**

1. Kilgour AHM, Rutherford M, Higson J, et al. Barriers and motivators to undertaking physical activity in adults over 70—a systematic review of the quantitative literature. *Age Ageing*. 2024;53(4):afae080. doi:10.1093/ageing/afae080

2. Physical Activity and Older Adults Systematic Literature Review.

3. Allen ME, Irizarry T, Einhorn J, et al. SMS-facilitated home blood pressure monitoring: A qualitative analysis of resultant health behavior change. *Patient Educ Couns*. 2019;102(12):2246-2253. doi:10.1016/j.pec.2019.06.015

4. Suffoletto B, Calabria J, Ross A, Callaway C, Yealy DM. A mobile phone text message program to measure oral antibiotic use and provide feedback on adherence to patients discharged from the emergency department. *Acad Emerg Med*. 2012;19(8):949-958. doi:10.1111/j.1553-2712.2012.01411.x

5. Senkowski V, Gannon C, Branscum P. Behavior Change Techniques Used in Theory of Planned Behavior Physical Activity Interventions Among Older Adults: A Systematic Review. *J Aging Phys Act*. 2019;27(5):746-754. doi:10.1123/japa.2018-0103

6. Knittle K, Nurmi J, Crutzen R, Hankonen N, Beattie M, Dombrowski SU. How can interventions increase motivation for physical activity? A systematic review and meta-analysis. *Health Psychol Rev*. 2018;12(3):211-230. doi:10.1080/17437199.2018.1435299

7. Deci EL, Ryan RM. Self-determination theory. In: *Handbook of Theories of Social Psychology, Vol. 1*. Sage Publications Ltd; 2012:416-436. doi:10.4135/9781446249215.n21

8. Teixeira PJ, Carraça EV, Markland D, Silva MN, Ryan RM. Exercise, physical activity, and self-determination theory: a systematic review. *Int J Behav Nutr Phys Act*. 2012;9:78. doi:10.1186/1479-5868-9-78

9. Tudor-Locke C, Craig CL, Aoyagi Y, et al. How many steps/day are enough? For older adults and special populations. *Int J Behav Nutr Phys Act*. 2011;8:80. doi:10.1186/1479-5868-8-80

10. Hart TL, Swartz AM, Cashin SE, Strath SJ. How many days of monitoring predict physical activity and sedentary behaviour in older adults? *Int J Behav Nutr Phys Act*. 2011;8:62. doi:10.1186/1479-5868-8-62

11. Stevens JA, Phelan EA. Development of STEADI: a fall prevention resource for health care providers. *Health Promot Pract*. 2013;14(5):706-714. doi:10.1177/1524839912463576

12. EuroQol Group. EuroQol--a new facility for the measurement of health-related quality of life. *Health Policy*. 1990;16(3):199-208. doi:10.1016/0168-8510(90)90421-9

13. Stewart AL, Mills KM, King AC, Haskell WL, Gillis D, Ritter PL. CHAMPS physical activity questionnaire for older adults: outcomes for interventions. *Med Sci Sports Exerc*. 2001;33(7):1126-1141. doi:10.1097/00005768-200107000-00010

14. Moschny A, Platen P, Klaassen-Mielke R, Trampisch U, Hinrichs T. Barriers to physical activity in older adults in Germany: a cross-sectional study. *Int J Behav Nutr Phys Act*. 2011;8:121. doi:10.1186/1479-5868-8-121

15. Norris M, Anderson R, Motl RW, Hayes S, Coote S. Minimum number of days required for a reliable estimate of daily step count and energy expenditure, in people with MS who walk unaided. *Gait Posture*. 2017;53:201-206. doi:10.1016/j.gaitpost.2017.02.005
